# A Randomized Trial of Azithromycin Versus Amoxicillin for the Treatment of *Chlamydia trachomatis* in pregnancy

**DOI:** 10.1155/S1064744901000321

**Published:** 2001

**Authors:** Jennifer Kacmar, Emily Cheh, Andrea Montagno, Jeffrey F. Peipert

**Affiliations:** Division of Research Department of Obstetrics and Gynecology Women and Infants Hospital 101 Dudley Street Providence RI 02905 USA

## Abstract

*Objective:* To compare the compliance, side effects and efficacy of amoxicillin and azithromycin for the treatment of *Chlamydia trachomatis* infection in pregnancy.

*Methods:* This is a randomized single-blind trial of women diagnosed with *C. trachomatis* before 33 weeks gestation.Women were randomly assigned either 500 mg amoxicillin orally three times per day for 7 days or a single dose of 1 g azithromycin orally. Patientswere interviewed by telephone approximately 3–7 days following therapy to assess compliance and side effects. Test of cure was performed at a follow-up visit 4–6 weeks following completion of therapy.

*Results:* Thirty-nine patients were randomized with 19 receiving amoxicillin and 20 receiving azithromycin. There were no differences in baseline data between the two groups, and there were no statistically significant differences in side effects, compliance or efficacy. In the amoxicillin group 84% of women took all pills, while 100% completed the single 1 g dose of azithromycin. Side effects were common in both groups (38% overall), with 40% of the azithromycin group reporting moderate to severe gastrointestinal side effects compared to 17% in the amoxicillin group (*p*  = 0.11). Of patients who returned for follow-up test of cure, 3 of 15 (20%) in the amoxicillin group were positive compared with 1 of 19 (5%) in the azithromycin group (*p*  = 0.3).

*Conclusions:* Side effects of therapy for *C. trachomatis* in pregnancy are common. Amoxicillin was slightly better tolerated than azithromycin. Compliance and cure rates with both regimens was high.


*Infect Dis Obstet Gynecol 2001;9:197–202*

### A randomized trial of azithromycin versus amoxicillin for the treatment of *Chlamydia trachomatis* in pregnancy

**Jennifer Kacmar, Emily Cheh, Andrea Montagno and Jeffrey F. Peipert**

*Division of Research, Department of Obstetrics and Gynecology, Women and Infants Hospital, Brown University Medical School, Providence, RI*

***Objective:*** To compare the compliance, side effects and efficacy of amoxicillin and azithromycin for the treatment of *Chlamydia trachomatis* infection in pregnancy.
***Methods:*** This is a randomized single-blind trial of women diagnosed with *C. trachomatis* before 33 weeks gestation. Women were randomly assigned either 500 mg amoxicillin orally three times per day for 7 days or a single dose of 1 g azithromycin orally. Patients were interviewed by telephone approximately 3–7 days following therapy to assess compliance and side effects. Test of cure was performed at a follow-up visit 4–6 weeks following completion of therapy.
***Results:*** Thirty-nine patients were randomized with 19 receiving amoxicillin and 20 receiving azithromycin. There were no differences in baseline data between the two groups, and there were no statistically significant differences in side effects, compliance or efficacy. In the amoxicillin group 84% of women took all pills, while 100% completed the single 1 g dose of azithromycin. Side effects were common in both groups (38% overall), with 40% of the azithromycin group reporting moderate to severe gastrointestinal side effects compared to 17% in the amoxicillin group ($p = 0.11$). Of patients who returned for follow-up test of cure, 3 of 15 (20%) in the amoxicillin group were positive compared with 1 of 19 (5%) in the azithromycin group ($p = 0.3$).
***Conclusions:*** Side effects of therapy for *C. trachomatis* in pregnancy are common. Amoxicillin was slightly better tolerated than azithromycin. Compliance and cure rates with both regimens was high.

Key words: CERVICITIS, CHLAMYDIA, THERAPY, ANTIBIOTICS, CLINICAL TRIAL

### INTRODUCTION

*Chlamydia trachomatis* is one of the most common sexually transmitted diseases (STDs) in the United States, with a prevalence of 2–24% in pregnant women[1,2]. Maternal infection with chlamydia has been associated with preterm rupture of membranes, preterm delivery, and delayed postpartum endometritis[3]. Risks to the neonate include pneumonia and conjunctivitis[4].

The 1998 Centers for Disease Control (CDC) treatment guidelines for STDs recommend erythromycin (500 mg orally four times per day for 7 days) or amoxicillin (500 mg orally three times per day for 7 days) as the treatments of choice for chlamydia in pregnancy[5]. It has been well established that gastrointestinal side-effects are common with erythromycin use (15–100%). Severe side effects have resulted in non-compliance with this therapy at rates of 12–33%. It has been suggested that amoxicillin is preferable to erythromycin because it is more easily tolerated, has comparable cure rates and is inexpensive[6–10].

Supported in part by NIH grant K24 HD01298-02, *Midcareer Investigator Award in Womens Health Research* from the National Institute of Child Health and Human Development.
Correspondence to: Jeffrey F. Peipert, Division of Research, Women and Infants Hospital, 101 Dudley Street, Providence, RI 02905. E-mail: jpeipert@wihri.org

Currently, azithromycin is recommended only as an alternative agent for treatment of chlamydia in pregnancy[5]. This may be due in part to cost (approximately $20–35 for a course of treatment), and because there are relatively few studies comparing it with other antibiotics in pregnant women. In fact, the CDC has stated that data are insufficient to recommend the routine use of azithromycin in pregnant women[5]. In practise, however, azithromycin is often used as a first-line therapy. For example, the state of Rhode Island has a program in which all indigent pregnant patients diagnosed with chlamydial infection are treated with azithromycin. Azithromycin is reported to have excellent cure rates, minimal side effects, and compliance can be virtually ensured given the one-time dosing regimen[10–12].

Based on our Medline review of the literature, we could find only one randomized trial comparing azithromycin with amoxicillin to treat chlamydia in pregnancy[13]. The study showed similar efficacy for these two agents. More women were intolerant of azithromycin (10.9%) than amoxicillin (5.5%), but the difference was not statistically significant ($p = 0.3$). The purpose of this study was to examine the side-effect profile, compliance and efficacy of azithromycin and amoxicillin in the treatment of *C. trachomatis* infection during pregnancy. We expected to find no difference in the test of cure between the two antibiotic treatment groups. However, we hypothesized that there would be a greater number of side effects and decreased compliance in the amoxicillin group compared with the azithromycin group.

### SUBJECTS AND METHODS

### Study Population

Prior to the initiation of the study, the Institutional Review Board approved the protocol and recruitment technique. The site of recruitment for this trial was the Women and Infants Hospital prenatal clinic located in the Women's Primary Care Center. We invited all pregnant women identified with *C. trachomatis* infection to participate. Routine chlamydia screens using ligase chain reaction (LCR) (Abbott Laboratories, Abbott Park, IL) are performed on all patients attending the prenatal clinic. Recruitment criteria included: positive test for chlamydia prior to 33 weeks gestation, ability to understand English, and willingness to give informed consent for participation. Exclusion criteria included other infections requiring antibiotic therapy (for example *Neisseria gonorrhoeae* or symptomatic vaginitis), known allergy or sensitivity to either amoxicillin or azithromycin, or gestational age greater that 33 weeks.

### Treatment

After informed consent was obtained, participants were asked to complete a short questionnaire to obtain baseline demographic characteristics. Randomization was performed using a random number sequence and opaque envelopes for concealment. Patients were randomized to receive either azithromycin 1 g orally as a single dose, or amoxicillin 500 mg orally three times per day for 7 days. Medications were provided free of charge to patients in both groups. A referral for treatment was given to all partners of patients testing positive for chlamydia, and patients were instructed to abstain from sexual intercourse until treatment was completed. If this was not possible, patients were encouraged to use condoms consistently and correctly to avoid reinfection.

Approximately 3–7 days post-therapy, a telephone interview with each patient was conducted to ascertain side effects and compliance with therapy. The post-therapy questionnaire asked about gastrointestinal symptoms (e.g. abdominal pain, nausea, vomiting or diarrhea), rash and other symptoms. Subjects were asked to rate their side effects as none, mild, moderate or severe. Participants who did not finish all their pills were asked how many pills were remaining. Approximately 4–6 weeks after treatment, a test of cure for chlamydia using LCR was obtained at a regularly-scheduled prenatal visit.

Prior to beginning the study, a sample-size calculation was performed. In order to detect a 20% difference in efficacy between amoxicillin and azithromycin with a type I error rate ($\alpha$) of 0.05 and a type II error rate ($\beta$) of 0.2 (power of 80%), 50 patients were needed for each treatment group.

However, due to time limitations and difficulties with recruitment, only 39 patients were enrolled in this trial.

We performed an intent-to-treat analysis, and did not omit subjects due to non-compliance or lack of partner treatment. Continuous data were analyzed using the unpaired, two-tailed Student *t*-test. Categorical variables were analyzed with chi-squared and Fischer exact test where appropriate. Non-parametric tests were used to evaluate the side-effect data that were graded on an ordinal scale. We used the binomial distribution to place 95% confidence intervals (CI) around proportions (e.g. cure rates, side effects, etc.).

### RESULTS

Between November 1998 and May 2000, 39 patients were enrolled and randomized. The mean age of the total population was 21.4 years (standard deviation (SD) = 5.7), the median gravidity was 2 (range 1–12), and the median parity was 0 (range 0–4). The median gestational age at enrollment was 12 weeks with a range of 5–31 weeks. In the study 46% of the patients were Hispanic, 33% Black, 15% Caucasian and 5% Asian. The two groups (amoxicillin and azithromycin) did not differ in terms of race, age, gravidity, parity or gestational age at enrollment (Table 1).

Of the 39 patients randomized, five failed to return for follow-up test of cure (four in the amoxicillin group and one in the azithromycin group). Of the 15 patients in the amoxicillin group who returned for a test of cure, three (20%) had positive tests of cure, while one of the 19 patients (5.3%) in the azithromycin group who returned had a positive test of cure ($p = 0.3$). Thus, the cure rate in the amoxicillin group was 80% (95% CI: 51.9–95.7), compared with 94.7% (95% CI: 74.0–99.9) in the azithromycin group. The one positive test of cure in the azithromycin group was in a patient who did not refer her partner for treatment, continued to have sexual intercourse and did not use a condom as recommended. The three positive tests of cure in the amoxicillin group were in women who reported no sexual activity since treatment.

In terms of compliance with therapy, all women in the azithromycin group took the single dose as directed (100% compliance). Three of 39 patients (7.7% of total; 15.8% of amoxicillin group) had pills left at the time of the follow-up phone contact. One patient had a single pill remaining, while two patients had two pills remaining. The three patients with positive tests of cure in the amoxicillin group reported taking all their pills. In terms of compliance with recommendations during therapy, only 25 of 39 patients (64%, 95% CI: 47.2–78.8) stated that their partner was treated for chlamydia since the patient's diagnosis was established.

When patients were asked whether they experienced any side effects or 'bad reactions' to the medication, 15 of 39 (38%, 95% CI: 23.4–55.4) responded affirmatively. Thirty-six per cent experienced nausea, 38% experienced vomiting, 18% experienced diarrhea and 15% complained of abdominal pain. Only nausea was statistically associated with therapy in the first trimester ($p = 0.04$).

**Table 1** Demographic and reproductive characteristics of patients by treatment group

| | *Amoxicillin* ($n$ = 19) | *Azithromycin* ($n$ = 20) |
|---|---|---|
| Ethnic origin | | |
| Caucasian | 3 (15%) | 3 (15%) |
| Black | 7 (37%) | 6 (30%) |
| Hispanic | 9 (47%) | 9 (45%) |
| Asian | 0 (0%) | 2 (10%) |
| Mean age, years (SD) | 21.7 (6.4) | 21.2 (5.1) |
| Median gravidity (range) | 2 (1–12) | 2 (1–6) |
| Median parity (range) | 0 (0–3) | 0 (0–4) |
| Mean GA at enrollment, weeks (SD) | 13.6 (8.0) | 14.8 (7.0) |

SD, standard deviation; GA, gestational age

When stratified by treatment group, 52.6% (95% CI: 28.9–75.6) of patients given azithromycin reported a reaction or side effect, while 29.4% (95% CI: 10.3–55.6) in the amoxicillin group had side effects ($p = 0.16$). The specific side effects reported are presented in Table 2, stratified by treatment group. In general, gastrointestinal side effects were very common, somewhat more so in the azithromycin group. For example, nausea was reported in 45% of azithromycin patients and 28% of amoxicillin patients. Abdominal pain was noted in 26% of azithromycin patients and 6% of amoxicillin patients. However, due to small sample size the differences in nausea and abdominal pain were not statistically significant. Of the azithromycin patients, 40% experienced moderate to severe gastrointestinal complaints, while 17% of amoxicillin patients had moderate to severe side-effects ($p = 0.11$). No patients had side effects severe enough to warrant change of medication. We found no relationship between gastrointestinal complaints post-therapy and reported symptoms pre-therapy. There were no significant immediate allergic reactions; however one woman in the amoxicillin group experienced a rash.

### DISCUSSION

Our hypothesis was that there is no difference in efficacy between azithromycin and amoxicillin. The three patients with positive tests of cure were all in the amoxicillin group. All three denied sexual intercourse after treatment. Thus, the cure rate in this group was 80%. We found one positive test of cure in the azithromycin group in a woman who stated she had unprotected intercourse with her partner who was not treated. Thus, this could easily be a reinfection rather than a medication failure. The cure rate in the azithromycin group was 95%. Our study was underpowered to detect a difference in cure rates based on these estimates. Given the small sample sizes of the trials performed to date, one cannot rule out that a difference in efficacy exists. A properly powered study is needed to provide additional evidence. However, our results corroborate other studies that showed no difference in efficacy between erythromycin, amoxicillin and azithromycin[11–14].

As expected, compliance with azithromycin was quite high. Based on self-report, compliance with a 7 day course of amoxicillin was also high. In our study, no patients reported missing more than two pills. Adair and colleagues[12] reported 98%

**Table 2** Reported side effects by treatment group

| | *Amoxicillin n/total** | *Azithromycin n/total** | p *Value* |
|---|---|---|---|
| Any reaction or side effect | 5/17 (29.4%) | 10/19 (52.6%) | 0.16 |
| Nausea | | | |
| Any | 5/18 (27.8%) | 9/20 (45%) | 0.27 |
| Moderate–severe | 2/18 (11.1%) | 4/20 (20%) | 0.45 |
| Vomiting | | | |
| Any | 6/18 (33.3%) | 9/20 (45%) | 0.46 |
| Moderate–severe | 3/18 (16.7%) | 4/20 (20%) | 0.79 |
| Diarrhea | | | |
| Any | 2/18 (11.1%) | 5/19 (26.3%) | 0.24 |
| Moderate–severe | 0/18 (0%) | 3/19 (15.8%) | 0.23 |
| Abdominal pain | | | |
| Any | 1/18 (5.6%) | 5/19 (26.3%) | 0.18 |
| Moderate–severe | 0/18 (0%) | 3/19 (15.8%) | 0.23 |
| GI Side effect | | | |
| Any | 10/18 (55.6%) | 13/20 (65%) | 0.55 |
| Moderate–severe | 3/18 (16.7%) | 8/20 (40%) | 0.11 |
| Other reactions | | | |
| Rash | 1/18 (5.6%) | 0 (0%) | 0.49 |

*Totals vary due to missing data. GI, gastrointestinal compliance with azithromycin compared with 54% for erythromycin. Compliance was defined by Adair and colleagues as strict adherence to prescription directions.

We were very surprised that the side effects with either regimen were so common. Close to 40% of patients experienced adverse reactions to the medication. Moderate to severe gastrointestinal side effects occurred in 40% of azithromycin patients, while 17% of amoxicillin patients experienced moderate to severe reactions. While this difference in our study was not statistically significant, a two-fold increase in side effects may be clinically significant. Adair and colleagues reported gastrointestinal side effects in 12% of patients taking azithromycin[12], but side effects were determined by patients' self-reports rather than systematic questioning. Wehbeh and co-workers reported that 7.4% of patients receiving azithromycin experienced side effects severe enough to warrant a change in medication[15]. In contrast, in a randomized trial of azithromycin versus erythromycin, Bush and Rosa[11] noted five of 15 subjects taking erythromycin were intolerant of the regimen compared to none of 15 in the azithromycin group. Despite the relatively common occurrence of side effects in our study, reactions were not severe enough to require change of therapeutic regimen.

The strengths of this study include the randomized design and a head-to-head comparison of two commonly used therapeutic regimens for *C. trachomatis* infection in pregnancy. The major limitation of this study is the small sample size. As a result, we had limited ability to detect clinically important differences in efficacy and adverse reactions and a greater chance for type II error. The small number of subjects results in wide confidence intervals and a lack of precision in our estimates. A final limitation is that on generalization or external validity. Our results may not apply to all populations with very different baseline characteristics.

In a survey of office-based obstetric-gynecologic practitioners[16], McGregor and colleagues reported that azithromycin was the preferred treatment for *C. trachomatis* infection during pregnancy. However, a Cochrane review of the literature regarding chlamydia treatment in pregnancy[10] questions how well the safety of azithromycin use in pregnancy has been established. In terms of costs, amoxicillin is the less expensive alternative. Amoxicillin tablets cost approximately \$0.31 per 500 mg tablet (total dose for three times a day regimen for 7 days = \$6.51) while azithromycin tablets cost approximately \$6.50 per 250 mg tablet (total 1 g dose = \$26). In a decision analysis evaluating antibiotic selection for *C. trachomatis* in pregnancy, Hueston and Lenhart[17] reported that the lowest failure rates could be achieved with the use of amoxicillin followed by azithromycin for treatment failures. Costs of this regimen were approximately 15% lower than starting with azithromycin. Additional studies with larger numbers of patients treated for *C. trachomatis* in pregnancy are necessary to determine the preferred and most cost-effective treatment regimen.

### REFERENCES

1. Centers for Disease Control and Prevention. Summary of notifiable diseases, United States, 1998. *Morbid Mortal Weekly Rep* 1999;47(53):1–93
2. Centers for Disease Control and Prevention. Recommendations for the prevention and treatment of *Chlamydia trachomatis* infections. *Morbid Mortal Weekly Report* 1993;42(RR-12):1–39
3. Andrews WA, Goldenberg RL, Mercer B, *et al.* The preterm prediction study: association of second trimester genitourinary chlamydia infection with subsequent spontaneous preterm birth. *Am J Obstet Gynecol* 2000;183(3):6662–668
4. Schachter J, Grossman M. Chlamydia. In Remington JS, Klein JO (eds.) *Infectious diseases of the fetus and newborn infant*. Philadelphia: WB Saunders 1995
5. Centers for Disease Control and Prevention. 1998 guidelines for treatment of sexually transmitted diseases. *Morbid Mortal Weekly Rep* 1998;47 (RR-1):80–1
6. Bush MR, Rosa C. Azithromycin and erythromycin in the treatment of cervical chlamydial infection during pregnancy. *Obstet Gynecol* 1994; 84:61–3

7. Crombleholme WR, Schacter J, Grossman M, *et al.* Amoxicillin therapy for *Chlamydia trachomatis* in pregnancy. *Obstet Gynecol* 1990;75:752–6
8. Magat AH, Alger LS, Nagey DA, *et al.* Double-blind randomized study comparing amoxicillin and erythromycin for the treatment of *Chlamydia trachomatis* in pregnancy. *Obstet Gynecol* 1993;81: 745–9
9. Silverman N, Sullivan M, Hochman M, *et al.* A randomized, prospective trial comparing amoxicillin and erythromycin for the treatment of *Chlamydia trachomatis* in pregnancy. *Infect Dis Obstet Gynecol* 1994;170:829–32
10. Brocklehurst P, Rooney G. Interventions for treating genital *Chlamydia trachomatis* infection in pregnancy (Cochrane review). *The Cochrane Library* 2000; Issue 4. Oxford: update software
11. Bush MR, Rosa C. Azithromycin and erythromycin in the treatment of cervical chlamydial infection during pregnancy. *Obstet Gynecol* 1994; 84:61–3
12. Adair CD, Gunter M, Stovall TG, *et al.* Chlamydia in pregnancy: a randomized trial of azithromycin and erythromycin. *Obstet Gynecol* 1998;91:165–8
13. Jacobson GF, Autry AM, Kirby RS, *et al.* A randomized controlled trial comparing amoxicillin and azithromycin for the treatment of *Chlamydia trachomatis* in pregnancy. *Am J Obstet Gynecol* 2001; 184:1352–6
14. Miller JM, Martin DH. Treatment of *Chlamydia trachomatis* infection in pregnant women. *Drugs* 2000;60:597–605
15. Wehbeh HA, Ruggeirio RM, Shahem S, *et al.* Single-dose azithromycin for *Chlamydia* in pregnant women. *J Reprod Med* 1998;43(6):509–14
16. McGregor JA, Hager WD, Gibbs RS, *et al.* Assessment of office-based care of sexually transmitted diseases and vaginitis and antibiotic decision-making by obstetrician-gynecologists. *Infect Dis Obstet Gynecol* 1998:6:247–51
17. Hueston WJ, Lenhart JG. A decision analysis to guide antibiotic selection for chlamydia infection during pregnancy. *Arch Fam Med* 1997;6:551–5

RECEIVED 05/30/01; ACCEPTED 10/05/01

## References

[PDF_00485] Adair C. D., Gunter M., Stovall T. G., McElroy G., Veille J. C., Ernest J. M. (1998). Chlamydia in pregnancy: a randomized trial of azithromycin and erythromycin.. Obstet Gynecol.

[PDF_00445] Andrews W. W., Goldenberg R. L., Mercer B., Iams J., Meis P., Moawad A., Das A., Vandorsten J. P., Caritis S. N., Thurnau G. (2000). The Preterm Prediction Study: association of second-trimester genitourinary chlamydia infection with subsequent spontaneous preterm birth.. Am J Obstet Gynecol.

[PDF_00481] Bush M. R., Rosa C. (1994). Azithromycin and erythromycin in the treatment of cervical chlamydial infection during pregnancy.. Obstet Gynecol.

[PDF_00458] Bush M. R., Rosa C. (1994). Azithromycin and erythromycin in the treatment of cervical chlamydial infection during pregnancy.. Obstet Gynecol.

[PDF_00464] Crombleholme W. R., Schachter J., Grossman M., Landers D. V., Sweet R. L. (1990). Amoxicillin therapy for Chlamydia trachomatis in pregnancy.. Obstet Gynecol.

[PDF_00504] Hueston W. J., Lenhart J. G. (1997). A decision analysis to guide antibiotic selection for Chlamydia infection during pregnancy.. Arch Fam Med.

[PDF_00488] Jacobson G. F., Autry A. M., Kirby R. S., Liverman E. M., Motley R. U. (2001). A randomized controlled trial comparing amoxicillin and azithromycin for the treatment of Chlamydia trachomatis in pregnancy.. Am J Obstet Gynecol.

[PDF_00467] Magat A. H., Alger L. S., Nagey D. A., Hatch V., Lovchik J. C. (1993). Double-blind randomized study comparing amoxicillin and erythromycin for the treatment of Chlamydia trachomatis in pregnancy.. Obstet Gynecol.

[PDF_00499] McGregor J. A., Hager W. D., Gibbs R. S., Schmidt L., Schulkin J. (1998). Assessment of office-based care of sexually transmitted diseases and vaginitis and antibiotic decision-making by obstetrician-gynecologists.. Infect Dis Obstet Gynecol.

[PDF_00493] Miller J. M., Martin D. H. (2000). Treatment of Chlamydia trachomatis infections in pregnant women.. Drugs.

[PDF_00472] Silverman N. S., Sullivan M., Hochman M., Womack M., Jungkind D. L. (1994). A randomized, prospective trial comparing amoxicillin and erythromycin for the treatment of Chlamydia trachomatis in pregnancy.. Am J Obstet Gynecol.

[PDF_00496] Wehbeh H. A., Ruggeirio R. M., Shahem S., Lopez G., Ali Y. (1998). Single-dose azithromycin for Chlamydia in pregnant women.. J Reprod Med.

